# Outcomes of Allograft Medial Patellofemoral Ligament Reconstruction in Patients Under 18: A Retrospective Cohort Study

**DOI:** 10.7759/cureus.90258

**Published:** 2025-08-16

**Authors:** Javier I Gonzalez, Maximiliano Espinosa, Gonzalo De La Fuente, Rodrigo Donoso, Hector Cifuentes

**Affiliations:** 1 Orthopaedic Surgery, Clinica Alemana de Santiago, Santiago, CHL; 2 Orthopaedic Surgery, Hospital Clínico San Borja Arriarán, Santiago, CHL

**Keywords:** adolescent knee, allograft, mpflr, patella alta, patellar instability, pediatric knee, pediatric orthopedics

## Abstract

Introduction: Patellar instability in pediatric patients often requires surgical stabilization. Allograft use in medial patellofemoral ligament reconstruction (MPFLR) remains controversial due to concerns derived from anterior cruciate ligament (ACL) literature.

Methods: We conducted a retrospective cohort study including 35 patients under 18 years old who underwent MPFLR with allografts between 2019 and 2022. The primary outcome was recurrent dislocation. Secondary outcomes included reoperations and complications.

Results: One (2.8%) patient had a recurrent dislocation. Six (17.1%) patients experienced complications, with only one requiring revision surgery.

Conclusion: Allograft MPFLR appears to be a safe and effective option for patellar instability in pediatric patients, with a low rate of failure and complications.

## Introduction

Recurrent patellar instability represents a significant orthopedic concern in pediatric patients, often necessitating surgical intervention to restore stability and mitigate the risk of recurrent dislocation [1,2]. Medial patellofemoral ligament reconstruction (MPFLR) is the current standard procedure for addressing patellar dislocation [3], including or not additional procedures in individuals presenting with predisposing anatomical abnormalities, such as patella alta, increased tibial tubercle-trochlear groove (TT-TG) distance, and genu valgum [4,5] in what is known as treatment a la carte [6]. Current technique uses autograft harvested from hamstring tendons (gracilis or semitendinosus), which have shown consistent good and excellent results regarding outcomes such as return to sports, re-dislocation rates, and patient-reported outcomes (PROs) [7], but is usually associated with donor site morbidity, including higher pain scores postoperatively [8].

Historically, the utilization of allografts in ligamentous reconstructions around the knee, especially in pediatric populations, has been associated with elevated complication rates, most notably in anterior cruciate ligament (ACL) reconstructions [9]. Complications commonly associated with the use of allografts in younger patients include high re-tear rates leading to a high reintervention risk, among others [10]. This is why the general consensus recommends against the use of allografts in ACL reconstructions; however, empirical evidence regarding the safety and efficacy of allografts in MPFLR remains scarce [2].

This study aims to evaluate the clinical outcomes of MPFLR employing allografts in patients under 18 years of age, with a specific focus on failure rates, reoperations, and postoperative complications. We hypothesize that allografts constitute a safe and efficacious alternative for MPFLR in the pediatric population.

## Materials and methods

A retrospective cohort study was conducted at our center, encompassing pediatric patients who underwent MPFLR with allografts between 2019 and 2022. Eligibility criteria included individuals younger than 18 years who had undergone surgical intervention for recurrent patellar dislocation. We excluded patients with prior patellar stabilization surgeries, systemic joint disorders, or inadequate follow-up data. Allografts used for reconstruction included the peroneus longus and semitendinosus tendons.

All MPFLR were performed using an anatomical technique with either peroneus longus or semitendinosus allografts. Femoral fixation was achieved with an interference screw, while patellar fixation was performed using two suture anchors. In skeletally immature patients with open physes, femoral tunnels were placed distal to the physis under fluoroscopic guidance to avoid growth plate injury, and in some cases, fixation was performed to the adductor tendon instead. No patients in this cohort presented with severe genu valgum requiring guided growth. Concomitant procedures, such as tibial tubercle osteotomy or lateral release, were performed when clinically indicated based on TT-TG distance or patellar tilt.

Data were collected from patient records and images by one orthopedic surgeon and one resident, and their findings were compared.

Demographic and clinical data, including age, sex, anatomical indices (Caton-Deschamps index, Insall-Salvati ratio, and TT-TG distance), and skeletal maturity indicators (presence of open physes), were meticulously documented. Surgical specifics, including graft type, fixation methodology, and concurrent procedures (e.g., tibial tubercle osteotomy and lateral release), were also recorded.

The primary outcome variable was failure rate, understood as recurrent postoperative patellar dislocation. Secondary outcomes included reoperation incidence and the occurrence of complications such as symptomatic hardware, overconstraint, and infection.

Descriptive analysis was employed to summarize patient characteristics and clinical outcomes. Categorical variables were expressed as percentages, while continuous variables were presented as medians and ranges. This study was approved by our institutional ethics committee. The authors declare no conflicts of interest, and there was no funding for this work.

This study adheres to the Strengthening the Reporting of Observational Studies in Epidemiology (STROBE) guidelines for reporting observational studies.

## Results

A total of 35 patients (12 male patients and 23 female patients) were included in the study cohort. The mean age at the time of surgery was 15.2 years (range: 11-17 years). The median follow-up duration was 13.5 months (range: 0.5-44 months) (Table 1).

**Table 1:** Demographic and Surgical Characteristics of the Study Population Note: Data are presented as numbers and percentages (number (%)) or mean ± SD (range), as appropriate. No statistical comparisons were performed, as this is a descriptive study. SD: standard deviation, ATT: anterior tibial tubercle

Characteristic	Subgroup	Statistical representation
Total patients included	35	Number
Sex: male	12 (32.4%)	Number (%)
Sex: female	23 (67.6%)	Number (%)
Age (years)	15.2 ± 1.7 (11-17)	Mean ± SD (range)
Follow-up (months)	13.2 ± 9.5 (0.5-44)	Mean ± SD (range)
Laterality: right	15 (42.8%)	Number (%)
Laterality: left	20 (57.1%)	Number (%)
Concurrent procedure: osteochondral fixation	3 (8.5%)	Number (%)
Concurrent procedure: ATT osteotomy	11 (31.4%)	Number (%)

Anatomical assessments (Table 2) revealed a mean Caton-Deschamps index of 1.23, with 20 (57.1%) patients diagnosed with patella alta (mean index: 1.36). The mean Insall-Salvati ratio was 1.19, and the mean TT-TG distance was 19.5 mm, with 17 (48.6%) patients exhibiting pathological TT-TG values. Six (17.14%) patients had open physes, indicative of skeletal immaturity, while nine (25.71%) patients presented with genu valgum. Skeletal maturity was assessed using left-hand and wrist radiographs, and lower limb alignment was evaluated with standing full-length lower limb radiographs.

**Table 2:** Anatomical Characteristics of the Study Population Note: Data are presented as numbers and percentages (number (%)) or mean ± SD, as appropriate. No statistical comparisons were performed, as this is a descriptive study. SD: standard deviation, TT-TG: tibial tubercle-trochlear groove

Characteristic	Value	Statistical representation
Patella alta	20 (57.1%)	Number (%)
Caton-Deschamps index	1.23 ± 0.20	Mean ± SD
Insall-Salvati index	1.19 ± 0.16	Mean ± SD
TT-TG distance	20 ± 3.44 mm	Mean ± SD
Altered TT-TG (>20 mm)	17 (48.5%)	Number (%)
Open physes	6 (17.14%)	Number (%)
Genu valgum	9 (25.71%)	Number (%)

Surgical outcomes and complications

Failure Rate

Only one (2.8%) patient experienced a recurrent postoperative patellar dislocation. No cases necessitated reoperation for residual instability. This patient was 13 years old, had open physes, and underwent isolated MPFLR with adductor tendon loop fixation.

Complications

Postoperative complications occurred in six (17.1%) patients. The most common was symptomatic hardware, reported in two (5.6%) cases. In both cases, the hardware corresponded to screws from concomitant tibial tubercle osteotomies. One patient developed overconstraint, necessitating revision surgery. No evidence of graft rupture, deep infection, or significant donor site morbidity were observed.

Reoperation Rate

Excluding the singular revision surgery for overconstraint, no additional reoperations were required.

## Discussion

The use of allografts in pediatric MPFLR has been a subject of clinical debate due to concerns regarding graft incorporation, immunogenic response, and long-term stability [11,12]. Prior studies focusing on ACL reconstruction in young patients have documented elevated failure rates associated with allografts, thereby engendering skepticism regarding their applicability in MPFLR [13-15]. However, the findings of the present study indicate that allografts can yield low failure and complication rates in MPFLR, presenting a viable alternative to autografts.

There are notable anatomical and structural distinctions between the ACL and the MPFL that likely account for this disparity [16]. Most notably, the ACL is located intra-articular, whereas the MPFL is situated extra-articular. The healing environments for intra-articular and extra-articular ligaments differ substantially [17], with the latter being generally more favorable for effective tissue repair. Consequently, the healing process after MPFLR may resemble that of a collateral ligament reconstruction more closely than that of a cruciate ligament [2].

The advantage of utilizing an allograft is primarily related to the potential complications associated with the donor site, including pain, infection, decreased flexor strength, and even neurological injury [8,18,19]. Nevertheless, certain studies have not demonstrated significant differences in these outcomes when comparing allografts to autografts [2].

Given the observed low recurrence rate of patellar dislocation (2.8%) and the absence of reoperations for instability, allograft MPFLR appears to be an effective surgical modality for pediatric patients with patellar instability. The relatively low complication rate further reinforces the safety profile of this approach. Surgeons may consider the utilization of allografts in scenarios where autograft harvesting is contraindicated or where patient preference necessitates an alternative grafting approach.

Several limitations warrant consideration. The retrospective study design inherently introduces selection and reporting biases. Furthermore, the relatively short follow-up period constrains the assessment of long-term clinical outcomes, such as late-stage graft failure, patellofemoral arthritis, or functional scores. Prospective, longitudinal studies with extended follow-up periods and larger sample sizes are requisite to substantiate these findings.

## Conclusions

Medial patellofemoral ligament reconstruction utilizing allografts constitutes a safe and efficacious intervention for pediatric patients presenting with patellar instability. The present study demonstrated a low failure rate (2.8%) and a minimal reoperation incidence, underscoring the clinical reliability of allograft MPFLR. While additional research is necessary to delineate long-term outcomes, the findings suggest that allografts represent a viable and advantageous alternative to autografts in pediatric MPFLR procedures.
